# Advances in the Management of Diabetes Mellitus: A Focus on Personalized Medicine

**DOI:** 10.7759/cureus.43697

**Published:** 2023-08-18

**Authors:** FNU Sugandh, Maria Chandio, FNU Raveena, Lakshya Kumar, FNU Karishma, Sundal Khuwaja, Unaib Ahmed Memon, Karoona Bai, Maham Kashif, Giustino Varrassi, Mahima Khatri, Satesh Kumar

**Affiliations:** 1 Medicine, Ghulam Muhammad Mahar Medical College, Sukkur, PAK; 2 Medicine, Civil Hospital Karachi, Karachi, PAK; 3 Medicine, Liaquat University of Medical and Health Sciences, Jamshoro, PAK; 4 General Medicine, Pandit Deendayal Upadhyay Medical College, Rajkot, IND; 5 Medical School, Jinnah Sindh Medical University, Karachi, PAK; 6 Neurology, Internal Medicine, Liaquat University of Medical and Health Sciences, Jamshoro, PAK; 7 Internal Medicine, Dow University of Health Sciences, Civil Hospital Karachi, Karachi, PAK; 8 Medicine, Khawaja Muhammad Safdar Medical College, Sialkot, PAK; 9 Pain Medicine, Paolo Procacci Foundation, Rome, ITA; 10 Medicine and Surgery, Dow University of Health Sciences, Karachi, PAK; 11 Medicine and Surgery, Shaheed Mohtarma Benazir Bhutto Medical College, Karachi, PAK

**Keywords:** review, medicine, management, mellitus, diabetes

## Abstract

Diabetes mellitus poses a substantial global health challenge, necessitating innovative approaches to improve patient outcomes. Conventional one-size-fits-all treatment strategies have shown limitations in addressing the diverse nature of the disease. In recent years, personalized medicine has emerged as a transformative solution, tailoring treatment plans based on individual genetic makeup, lifestyle factors, and health characteristics. This review highlights the role of genetic screening in predicting diabetes susceptibility and response to treatment, as well as the potential of pharmacogenomics in optimizing medication choices. Moreover, it discusses the incorporation of lifestyle modifications and behavioral interventions to empower patients in their health journey. Telemedicine and remote patient monitoring are also examined for their role in enhancing accessibility and adherence. Ethical considerations and challenges in implementing personalized medicine are addressed. The review envisions a future where personalized medicine becomes a cornerstone in diabetes management, ensuring improved patient outcomes and fostering more effective and patient-centric care on a global scale.

## Introduction and background

Diabetes mellitus, also referred to as diabetes, is a multifaceted and enduring metabolic disorder that has become a prominent global health concern. The global impact of this issue is significant, affecting a large population and presenting substantial health risks, thereby placing a considerable strain on healthcare systems [1]. Diabetes is a medical condition characterized by elevated blood glucose levels resulting from insufficient insulin production or impaired insulin function. This condition can lead to significant complications, affecting multiple organ systems and significantly reducing the overall quality of life for individuals affected by it. As we explore the intricate nature of diabetes and its widespread occurrence worldwide, the field of healthcare is witnessing the emergence of personalized medicine as a promising avenue. This innovative approach holds significant promise in revolutionizing diabetes management and improving patient outcomes. The incidence of diabetes has reached concerning proportions, rendering it a prominent global health issue [2]. According to the data provided by the International Diabetes Federation (IDF), the global population of adults diagnosed with diabetes in 2019 was estimated to be around 463 million. The number is anticipated to increase significantly, as projections suggest that by 2045, approximately 700 million individuals may be impacted if prompt action is not taken. The increase in diabetes cases can be attributed to a combination of factors, such as sedentary lifestyles, unhealthy dietary habits, escalating obesity rates, and a global aging population [3].

Diabetes is a condition that affects individuals across all age groups, races, and socioeconomic backgrounds without discrimination. Nevertheless, the disease’s impact is notably significant in low- and middle-income countries, where the availability of healthcare, education, and resources is constrained, thereby amplifying its severe repercussions. As a result, diabetes contributes to the persistence of health disparities, exacerbating variations in healthcare outcomes among different regions and populations [4]. In light of the growing diabetes epidemic, it has become evident that the conventional approach of employing a uniform strategy for managing the disease has demonstrated its inherent limitations. Diabetes is a complex medical condition characterized by a wide range of clinical manifestations and individual variations in treatment response. The utilization of standardized treatment regimens may not adequately cater to the individualized needs and unique characteristics of every patient, resulting in less-than-optimal outcomes and difficulties in attaining optimal glycemic control. In light of these challenges, the field of personalized medicine, also referred to as precision medicine or individualized medicine, has emerged as a highly promising solution [5]. Personalized medicine is a healthcare approach that customizes treatment plans and interventions based on individual patient characteristics. These characteristics encompass genetic makeup, lifestyle factors, environmental influences, and specific health data. By utilizing this personalized approach, healthcare providers can maximize treatment decisions, leading to improved treatment effectiveness and enhanced patient well-being [6].

A key aspect of personalized medicine in the management of diabetes is its capacity to elucidate the genetic factors that impact an individual’s vulnerability to the condition. The utilization of genetic screening facilitates the timely detection of individuals who may be susceptible to developing diabetes, thereby enabling the implementation of focused preventive interventions and adjustments to their lifestyle [7]. In addition, genetic insights provide valuable guidance to clinicians when it comes to choosing the most appropriate medications for individual patients. This helps to reduce the likelihood of adverse drug reactions and treatment non-responsiveness [8]. The field of personalized medicine also incorporates cutting-edge technologies, such as continuous glucose monitoring and wearable devices, which enable patients to actively engage in the management of their diabetes. By utilizing real-time data and receiving personalized feedback, patients are able to make well-informed decisions regarding their diet, exercise, and medication adherence [9]. This approach fosters a sense of empowerment and ownership over their health [10].

## Review

Diabetes mellitus overview

Diabetes mellitus is a multifaceted and enduring metabolic disorder that impacts a significant global population. The condition is characterized by increased levels of glucose in the bloodstream, referred to as hyperglycemia. This occurs due to insufficient production of insulin by the body or inadequate utilization of the insulin it produces [1]. Insulin is a hormone synthesized by the pancreas that plays a crucial role in the regulation of blood sugar levels and facilitates the uptake of glucose into cells, thereby enabling the provision of energy. When the normal functioning of this process is interrupted, it can result in various complications that have the potential to impact multiple organ systems within the body [1,2].

Types of Diabetes

Type 1 diabetes: Type 1 diabetes, also referred to as insulin-dependent or juvenile diabetes, is an autoimmune condition characterized by the erroneous targeting and destruction of the insulin-producing beta cells in the pancreas by the body’s immune system. Consequently, individuals diagnosed with type 1 diabetes exhibit minimal or no production of insulin and necessitate daily administration of insulin injections for their survival [2]. Typically, this condition manifests during childhood or adolescence; however, it may also present in adult individuals. The precise etiology of type 1 diabetes remains incompletely elucidated; however, it is postulated to arise from a confluence of genetic and environmental influences. Specific genes have been found to be linked to a heightened susceptibility to type 1 diabetes. However, it is important to note that an external stimulus, such as a viral infection, may be required to initiate the autoimmune response [3]. In individuals with type 1 diabetes, the immune system erroneously recognizes the beta cells located in the pancreas as foreign entities and initiates an immune response targeting these cells. The immune response in question entails the generation of autoantibodies that selectively target proteins located on the surface of beta cells. The ongoing destruction of beta cells results in a gradual decrease in insulin production, eventually leading to inadequate regulation of blood sugar levels [4].

Type 2 diabetes: Type 2 diabetes, also known as non-insulin-dependent diabetes, is the predominant type of diabetes, constituting approximately 90-95% of all cases of diabetes. Insulin resistance or insufficient insulin production in the body leads to the inability to maintain normal blood glucose levels. Type 2 diabetes is frequently linked to lifestyle factors such as obesity, sedentary behavior, and an unhealthy diet [2-4]. The prevalence of this condition is predominantly observed in adults; however, there has been a notable increase in the diagnosis of children and adolescents, which can be attributed to the growing issue of childhood obesity.

Pathophysiology and mechanism of type 2 diabetes: Type 2 diabetes is distinguished by the presence of insulin resistance, wherein the cells within the body fail to adequately respond to the physiological effects of insulin. In the beginning, the pancreas responds by increasing insulin production to counteract the resistance and sustain optimal levels of blood glucose. Over time, it is possible for the beta cells in the pancreas to experience exhaustion, resulting in a diminished capacity to produce sufficient insulin [5]. This, in turn, can lead to the occurrence of hyperglycemia. Obesity, specifically the presence of excessive abdominal fat, is a prominent risk factor associated with insulin resistance. Adipose tissue, also known as fat cells, releases inflammatory substances and hormones that have a negative impact on insulin signaling in cells. Furthermore, it is worth noting that genetic factors contribute to the predisposition of individuals to type 2 diabetes. However, it is important to acknowledge that the development of this condition is primarily influenced by lifestyle and environmental factors [6].

Gestational diabetes: Gestational diabetes is a condition that arises during pregnancy and impacts around 3-9% of expectant mothers. Gestational diabetes is a condition characterized by impaired insulin action caused by hormones produced by the placenta, resulting in elevated blood sugar levels. Gestational diabetes typically resolves postpartum; however, women who have been affected by this condition are at a heightened risk of developing type 2 diabetes in the future [5,6]. During the course of pregnancy, the placenta plays a vital role in the production of hormones that facilitate the growth and development of the baby. However, it is important to note that these hormones can potentially impede the normal functioning of insulin within the mother’s body. Certain hormones, such as human placental lactogen and progesterone, have the potential to induce insulin resistance, thereby diminishing the ability of maternal cells to take up glucose [6]. Consequently, blood glucose levels increase to supply sufficient nutrients to support the developing fetus. Typically, the maternal pancreas exhibits increased insulin production as a means of counteracting insulin resistance. However, in certain women, this compensatory mechanism proves to be inadequate, leading to the development of gestational diabetes.

The Global Burden of Diabetes

The global prevalence of diabetes has reached epidemic proportions, displaying a worrisome upward trend. Based on data from the IDF, it was estimated that in 2019, there were approximately 463 million adults globally who were diagnosed with diabetes [6]. The projected figure for this number is expected to reach 700 million by 2045 in the absence of effective prevention and management strategies. It is concerning to note that a significant proportion, exceeding 50%, of diabetes cases go undiagnosed, thereby intensifying the impact of this condition. Diabetes has a significant impact on individuals across various age groups, ethnicities, and socioeconomic statuses. However, the prevalence of diabetes is notably elevated in low- and middle-income countries, where individuals may face challenges in accessing adequate healthcare and resources for diabetes management. The ramifications of diabetes extend beyond personal health, imposing a significant economic burden on healthcare systems and societies at large [6,7].

Despite the considerable progress made in the field of diabetes management, there are still several challenges that continue to impede the effective control of this disease. The rising prevalence of type 2 diabetes can be primarily attributed to lifestyle changes, specifically the adoption of sedentary behavior and unhealthy dietary habits. The prevalence of obesity is a significant contributing factor that drives the emergence of insulin resistance and metabolic abnormalities. It is observed that a significant number of individuals with diabetes go undiagnosed, resulting in a delay in receiving appropriate treatment and an elevated likelihood of experiencing complications [7,8]. The importance of timely intervention and effective disease management necessitates the implementation of screening programs for early detection. Managing glycemic control is a considerable undertaking, particularly for individuals diagnosed with type 2 diabetes. Various factors, including medication adherence, dietary compliance, and physical activity, are crucial in effectively managing blood glucose levels. Variability among individuals with diabetes exhibits notable diversity in its clinical presentations and treatment responses. The conventional approach, which assumes that one solution fits all, may not sufficiently cater to the diverse needs and challenges experienced by patients. This highlights the significance of personalized medicine [9].

Diabetes is associated with an elevated risk of diverse complications, including cardiovascular disease, neuropathy, retinopathy, and nephropathy. The effective management of these comorbidities necessitates a comprehensive and interdisciplinary approach to diabetes care. The distribution of access to quality healthcare and resources for diabetes management is uneven, especially in low-resource settings [10]. Discrepancies in access can result in less-than-optimal healthcare and worsen disparities in health. The necessity for personalized approaches in diabetes management stems from the acknowledgment of individual variability and the distinct factors that influence the progression of diabetes and the response to treatment. Personalized medicine is a medical approach that customizes treatment plans according to an individual’s genetic profile, lifestyle, and health attributes. This method aims to enhance the effectiveness and precision of interventions [11].

Genetics and diabetes risk

Diabetes mellitus is a versatile metabolic condition that is distinguished by heightened levels of blood glucose caused by either inadequate insulin production or impaired insulin function. Lifestyle factors are known to have a significant impact on the development of diabetes [10,11]. However, it is important to acknowledge that genetics also plays a substantial role in determining an individual’s vulnerability to this disease. In recent years, significant progress has been made in the field of genetic research, providing valuable insights into the genetic factors that contribute to an elevated susceptibility to the development of diabetes. Gaining a comprehensive understanding of the genetic underpinnings of diabetes can have significant implications for various aspects such as risk prediction, early detection, and the development of personalized management strategies [10-12].

Genetic Factors Associated With an Increased Risk of Diabetes Development

Type 1 diabetes is a medical condition characterized by the body’s inability to produce insulin. Type 1 diabetes is predominantly recognized as an autoimmune disease, with genetic factors playing a substantial role in its onset. Certain human leukocyte antigen (HLA) genes, specifically *HLA-DR3* and *HLA-DR4*, have demonstrated a significant correlation with an elevated susceptibility to the development of type 1 diabetes [13]. These genes are crucial in the regulation of the immune system and are responsible for the identification of self and non-self-antigens. The presence of genetic variations in the *HLA* genes has been observed to have an impact on an individual’s immune response, rendering them more vulnerable to autoimmunity targeting pancreatic beta cells. Additional non-HLA genes, namely, *INS*, *PTPN22*, and *CTLA4*, have also been associated with the development of type 1 diabetes. These genes play a role in immune regulation and contribute to the disruption of immune tolerance toward beta cells, ultimately resulting in their destruction [14].

Type 2 diabetes is a complex condition that is influenced by a combination of genetic and environmental factors. Multiple genetic loci have been identified via genome-wide association studies (GWAS) that exhibit an elevated susceptibility to type 2 diabetes. It is worth mentioning that genes associated with beta-cell function, namely, *TCF7L2*, *KCNJ11*, and *HNF1A*, as well as genes linked to insulin resistance, such as *PPARG*, *IRS1*, and *GCKR*, have been implicated in this context [14]. The gene *TCF7L2 *is widely recognized as having the strongest association with the risk of developing type 2 diabetes. Genetic variations in the *TCF7L2 *gene have been found to impact insulin secretion and glucose metabolism, thereby playing a role in the development of impaired glycemic control.

Gestational diabetes is also influenced by genetic factors, although its study is not as extensive as that of type 1 and type 2 diabetes. There is evidence suggesting that genetic variants may play a role in the development of gestational diabetes by affecting insulin resistance, beta-cell function, and inflammation [15].

A multitude of genetic studies have significantly contributed to our comprehension of the genetic underpinnings associated with the development of diabetes. GWAS, which investigate genetic variants throughout the entire genome, have successfully identified numerous genetic loci that are associated with the risk of developing type 1 and Type 2 diabetes. The Type 1 Diabetes Genetics Consortium (T1DGC) has played a crucial role in the identification of *HLA *genes and other non-HLA genetic variants associated with susceptibility to type 1 diabetes. The research findings have shed light on the intricate relationship between genetic predisposition and environmental triggers in the pathogenesis of type 1 diabetes [15,16]. The Diabetes Genetics Replication and Meta-Analysis Consortium (DIAGRAM) and the Genetic Investigation of Anthropometric Traits (GIANT) have conducted extensive GWAS to identify specific genetic loci that are associated with an increased risk of type 2 diabetes. These studies have identified genes associated with beta-cell function, insulin resistance, and glucose metabolism as potential factors in the development of the disease [14-16].

The Significance of Genetic Testing and Risk Prediction in Patients With Diabetes

Early detection and prevention: Genetic testing for assessing the risk of diabetes can effectively identify individuals who are at a heightened risk of developing the disease. The timely identification of diabetes allows for the implementation of focused interventions, adjustments to one’s lifestyle, and the adoption of preventive measures to mitigate the risk of developing the condition [17]. For individuals at high risk, implementing lifestyle modifications, such as engaging in regular physical activity and achieving weight loss, has the potential to delay or even prevent the onset of type 2 diabetes.

Individualized treatment approaches: Genetic testing can inform tailored treatment strategies for individuals with diabetes. Genetic testing can be utilized in type 1 diabetes to identify individuals who may potentially benefit from immunomodulatory therapies aimed at preserving beta-cell function [18]. The utilization of genetic information in type 2 diabetes can assist in the selection of the most suitable medications to achieve optimal glycemic control.

Family screening and genetic counseling: Genetic testing can provide valuable insights for assessing the risk of diabetes in family members. The identification of high-risk family members can serve as a catalyst for encouraging them to undergo regular screening and adopt proactive preventive measures [14-18]. Genetic counseling plays a vital role in providing individuals with essential knowledge regarding their genetic risk, enabling them to make well-informed decisions concerning their health.

Precision medicine and drug development: The increasing comprehension of the genetic underpinnings of diabetes is propelling the progress of precision medicine strategies. The strategic focus on specific genetic pathways has the potential to pave the way for the creation of innovative treatments that are customized to an individual’s unique genetic profile [15,16].

Ethical considerations: The utilization of genetic testing to assess diabetes risk gives rise to ethical concerns pertaining to privacy, data security, and the potential for discriminatory practices. It is imperative to implement appropriate measures and ensure that individuals provide informed consent to effectively address these ethical considerations [12-15].

The elucidation of the genetic basis of diabetes has yielded valuable insights into the pathogenesis of the disease and the ability to predict risk. Genetic testing has the capability to identify individuals who are at a heightened risk of developing diabetes. This valuable information allows for the implementation of early interventions, targeted treatments, and preventive measures. Genetic factors are known to exert a substantial influence on the development of diabetes; however, it is important to recognize that diabetes is a multifaceted and intricate condition that is influenced by both genetic and environmental factors [17,18]. The incorporation of genetic information into diabetes management has the potential to yield more individualized and efficient strategies, thereby optimizing patient outcomes and bolstering our capacity to address the worldwide impact of diabetes mellitus. It is imperative to give thorough thought to ethical considerations and patient privacy to responsibly implement genetic testing in diabetes care. With ongoing advancements in genetics and diabetes research, personalized medicine has the potential to significantly transform diabetes management and enhance the quality of life for the large population impacted by this prevalent metabolic disorder [19].

Biomarkers and predictive models in diabetes mellitus

Biomarkers are quantifiable indicators that offer valuable insights into the current state of a disease and the effectiveness of treatment. Biomarkers have a substantial impact in the field of diabetes by serving as valuable tools for predicting disease progression, evaluating the effectiveness of treatments, and identifying individuals at heightened risk. Glycemic biomarkers, such as hemoglobin A1c (HbA1c), are crucial for monitoring disease progression as they provide valuable insights into long-term glycemic control [20]. Elevated levels of HbA1c are indicative of suboptimal glycemic control and an augmented susceptibility to complications associated with diabetes. Consistent monitoring of HbA1c assists healthcare professionals in making necessary adjustments to treatment plans to attain desired glycemic levels.

Biomarkers that assess beta-cell function, such as C-peptide, play a crucial role in predicting the progression of type 2 diabetes. C-peptide is a metabolic product derived from the production of insulin and is commonly used as an indicator of beta-cell functionality. Decreased levels of C-peptide indicate a progressive decline in beta-cell function and an increase in insulin resistance, thereby highlighting the necessity for more intensive treatment to maintain beta-cell function [21]. Inflammatory biomarkers, such as C-reactive protein (CRP) and interleukin-6 (IL-6), serve as indicators of the inflammatory state and have the potential to aid in predicting the likelihood of developing diabetes and experiencing cardiovascular complications. The implementation of targeted interventions aimed at managing inflammation has the potential to enhance diabetes outcomes and mitigate the likelihood of complications [22].

Genetic biomarkers, which are identified through the process of genetic testing, offer valuable insights into an individual’s genetic predisposition to diabetes as well as their response to treatment. Genetic variations linked to the risk of diabetes and drug metabolism can inform personalized treatment strategies, allowing for the customization of medications according to individual genetic profiles. Adipokines, which are released by adipose tissue, have a significant impact on insulin sensitivity and glucose metabolism [23]. Leptin and adiponectin are adipokines that have been linked to insulin resistance and an increased risk of developing diabetes. There is a correlation between elevated leptin levels and decreased adiponectin levels with the development of insulin resistance and diabetes. Predictive models leverage patient data, encompassing clinical, genetic, and lifestyle factors, to anticipate the progression of diseases and evaluate the effectiveness of treatments. These models assist healthcare providers in making well-informed decisions and customizing treatment plans to meet the specific needs of each patient. Risk scoring models utilize a comprehensive set of risk factors to assess and estimate an individual’s likelihood of developing diabetes or experiencing complications associated with diabetes [21-23]. These models facilitate the early detection, prevention, and implementation of targeted interventions to mitigate the progression of diseases.

Decision-support systems utilize advanced data analytics and machine learning techniques to efficiently analyze extensive patient data and produce tailored treatment recommendations. These systems have the capability to forecast glycemic responses to various medications and lifestyle interventions, thereby providing guidance for treatment selection to achieve optimal outcomes. Treatment response models are utilized to analyze patient data to make predictions regarding the individual’s response to particular medications and interventions [22,23]. These models assist in the identification of factors that are associated with treatment success or failure, thereby facilitating the selection of the most optimal treatment plan for individual patients. Continuous glucose monitoring (CGM) algorithms leverage real-time glucose data to forecast forthcoming glucose levels and trends. These algorithms facilitate proactive modifications to insulin doses and lifestyle adjustments, thereby promoting improved glycemic control and mitigating the risk of hypoglycemia and hyperglycemia. Artificial pancreas systems integrate CGM technology with insulin pump therapy, enabling the automated administration of insulin in response to real-time glucose data [15-18]. These closed-loop systems employ predictive algorithms to enhance glycemic control, thereby alleviating the burden of diabetes management for patients.

Precision medicine in monogenic diabetes involves the customization of treatment strategies based on specific genetic mutations that impact the functioning of beta cells and the production of insulin. Genetic testing has the capability to identify specific gene mutations that are accountable for monogenic diabetes. This enables a more accurate diagnosis and facilitates the implementation of personalized treatment approaches. The field of pharmacogenomics in type 2 diabetes research investigates the impact of genetic variations on the response to pharmaceutical interventions. Genetic testing has the capability to identify patients who may exhibit an augmented response to specific medications [20-22]. For example, individuals possessing specific genetic variants may exhibit enhanced responsiveness to metformin, which is widely recognized as the primary medication for managing type 2 diabetes. CGM offers real-time information regarding glucose levels, facilitating personalized adjustments to insulin therapy. The utilization of a data-driven approach enables the timely adjustment of insulin doses, resulting in the optimization of glycemic control and a reduction in the potential risk of hypoglycemia. The identification of gestational diabetes at an early stage can be achieved by utilizing biomarkers and predictive models. Implementing early intervention through lifestyle modifications or pharmacological treatments has the potential to effectively mitigate the risk of complications for both the mother and the fetus [18-20]. Risk-scoring models have demonstrated efficacy in the identification of individuals with a heightened susceptibility to the development of type 2 diabetes. Strategic interventions, such as implementing lifestyle modifications and enrolling in weight loss programs, have the potential to effectively delay or even prevent the onset of diabetes in individuals who are at high risk [20-22].

In summary, the utilization of biomarkers and predictive models has significantly transformed the field of diabetes management by offering valuable insights into the progression of the disease, response to treatment, and the development of personalized treatment strategies. Glycemic, beta-cell function, inflammatory, genetic, and adipokine biomarkers are essential factors in the prediction of diabetes development and treatment outcomes [21-23]. Predictive models leverage patient data to anticipate the progression of diseases and evaluate the effectiveness of treatments, thereby assisting healthcare providers in making well-informed decisions. Biomarker-driven treatment strategies, such as precision medicine in monogenic diabetes and pharmacogenomics in type 2 diabetes, have proven to be successful in enhancing the personalization and effectiveness of diabetes management. The integration of biomarkers and predictive models in diabetes care presents an opportunity to enhance patient outcomes, improve treatment effectiveness, and establish a more patient-centered and precise approach to managing diabetes [20-24].

Individualized glycemic control in diabetes mellitus

Personalized Glycemic Targets Based on Patient Characteristics and Comorbidities

The American Diabetes Association (ADA) and other international guidelines advocate for the use of personalized glycemic targets in the management of diabetes. The determination of glycemic targets considers a range of patient-specific factors, including age, duration of diabetes, presence of comorbidities (such as cardiovascular disease or kidney disease), and risk of hypoglycemia. Elderly individuals or those with notable comorbidities may require higher glycemic targets to minimize the risk of hypoglycemia [20-23]. Conversely, younger and healthier individuals may have more stringent glycemic goals. Personalized insulin therapy is of utmost importance in managing type 1 diabetes, as it allows for tailoring treatment to the individual’s lifestyle, dietary habits, and exercise regimen. This approach is essential for achieving optimal glycemic control. CGM devices are essential tools in personalized glycemic management, as they offer real-time information on blood glucose levels and assist in promptly adjusting insulin dosages [25].

Current Advancements and Innovations in Continuous Glucose Monitoring Technologies and Devices

The field of diabetes management has been significantly transformed by technological advancements, with a notable impact on CGM systems. The conventional method of fingerstick blood glucose testing has certain limitations when it comes to accurately capturing and tracking fluctuations and trends in glucose levels. CGM devices, in contrast, provide a continuous flow of glucose data, enabling a more comprehensive assessment of an individual’s glycemic profile [21-25]. The most recent CGM systems are equipped with smaller and more comfortable sensors that have the capability to be worn for extended periods of time. The sensors have the capability to transmit glucose data to smartphones or dedicated readers in an automated manner. This enables the provision of real-time information and alerts pertaining to high or low glucose levels. Certain CGM systems have the capability to seamlessly integrate with insulin pumps, facilitating the process of automated insulin delivery. This advanced functionality is commonly referred to as an artificial pancreas or closed-loop system [24]. The aforementioned advancements have had a substantial impact on enhancing glycemic control and alleviating the burden associated with diabetes management for patients.

The Efficacy of Personalized Glycemic Control in Enhancing Patient Outcomes

Multiple studies have provided evidence regarding the advantages of personalized glycemic control in the management of diabetes. Customizing glycemic targets based on the individual patient’s specific needs and circumstances has been linked to various favorable outcomes [25].

Minimized hypoglycemia risk: Customized glycemic control takes into consideration the potential risk of hypoglycemia, particularly in older individuals or those with significant concurrent medical conditions. The prevention of severe hypoglycemic events is associated with improved overall health and decreased hospitalizations [26].

Enhanced quality of life: The implementation of personalized glycemic targets, which account for the patient’s lifestyle and preferences, has been shown to lead to improved adherence to the treatment plan. This results in an improved quality of life and enhanced patient satisfaction in the management of diabetes [27].

Reduced risk of diabetes complications: Maintaining tight glycemic control has been linked to a diminished risk of complications associated with diabetes, including diabetic retinopathy, nephropathy, and neuropathy. The implementation of personalized glycemic control strategies plays a crucial role in the maintenance of stable blood glucose levels, thereby mitigating the potential risks associated with long-term complications [28].

Enhanced treatment adherence: Active patient involvement in establishing glycemic targets and treatment plans has been shown to increase adherence to prescribed therapies. The active involvement and empowerment of patients play a pivotal role in achieving effective diabetes management [27].

Decreased healthcare expenditures: Enhanced glycemic control achieved through personalized strategies has the potential to reduce hospitalizations and visits to emergency rooms, thereby generating cost savings for healthcare systems [27].

Challenges and Considerations in Personalized Glycemic Control

While the implementation of individualized glycemic control presents notable benefits, it is crucial to acknowledge and address various challenges and considerations.

Continuous monitoring compliance: The efficacy of personalized glycemic control depends on the precise and consistent glucose data acquired via continuous monitoring. It is imperative for patients to demonstrate a willingness to consistently utilize CGM devices and accurately interpret the data they provide [28].

Education and training: It is imperative for healthcare providers to deliver thorough education and training to patients regarding the proper utilization of CGM devices and the accurate interpretation of glucose data. It is crucial to comprehend the importance of glucose trends and to make appropriate insulin adjustments to achieve successful individualized glycemic control [29].

Access and affordability: CGM technology and devices may pose challenges in terms of accessibility and affordability for certain patients, particularly those in resource-limited settings. It is imperative to prioritize the establishment of equitable access to these technologies to facilitate their widespread adoption [30].

Psychosocial factors: Effective diabetes management encompasses more than just glucose control; it necessitates the consideration and management of psychosocial factors that can influence treatment adherence. Various factors, such as stress, depression, and social support, have the potential to impact a patient’s capacity to effectively manage diabetes [17].

The concept of individualized glycemic control entails a personalized and patient-centered strategy for the management of diabetes mellitus. Customizing glycemic targets and treatment plans according to patient characteristics, comorbidities, and lifestyle preferences has demonstrated efficacy in enhancing patient outcomes, mitigating complications, and improving quality of life. CGM technologies have been instrumental in personalized glycemic management, offering up-to-date information to guide treatment choices and enhance glycemic control [18]. Despite the existence of challenges, progress in diabetes technology and an increased emphasis on patient empowerment are creating a path toward a future where personalized glycemic control becomes the norm in healthcare. This transformation in diabetes management has the potential to significantly enhance the quality of life for the millions of individuals impacted by this enduring metabolic disorder.

Pharmacogenomics and diabetes medications

Pharmacogenomics, an interdisciplinary field that integrates the disciplines of pharmacology and genomics, assumes a pivotal role in customizing diabetes drug therapies to suit the unique needs of individual patients. Pharmacogenomics aims to utilize the analysis of genetic variations in an individual’s DNA to forecast their response to particular medications and potential adverse reactions [22]. The implementation of a personalized approach to diabetes treatment shows significant potential in enhancing the effectiveness of medications and reducing the occurrence of adverse effects, ultimately resulting in better patient outcomes.

Genetic Variations and Drug Response in Diabetes Medications

In recent years, there has been growing interest in understanding the impact of genetic variations on the response to diabetes medications. This area of research aims to elucidate the role of genetic factors in determining individual differences in drug efficacy and adverse reactions. By uncovering these associations, healthcare professionals can tailor treatment plans to optimize further effects. Genetic variations have a substantial impact on the metabolic processing and therapeutic response of diabetes medications in individuals [23]. Certain genetic factors can influence the process of drug metabolism, drug targets, and drug transporters, all of which can have a significant impact on the efficacy and safety of the drug. Genetic variations can result in diverse drug responses, encompassing a lack of response, improved effectiveness, or heightened susceptibility to adverse reactions [24].

Pharmacogenomics-Guided Diabetes Treatment Approaches

Metformin and the *OCT1 *gene: Metformin, a frequently prescribed initial treatment for type 2 diabetes, is transported into hepatic cells via the *OCT1*. Specific genetic variants of the *OCT1 *gene have the potential to cause a decrease in the transportation of metformin into the liver, resulting in a reduction in the effectiveness of the drug. The identification of patients with these genetic variants can assist clinicians in making appropriate adjustments to metformin dosages or selecting alternative medications to achieve improved treatment outcomes [12].

Sulfonylureas are known to elicit the release of insulin from pancreatic beta cells through their interaction with *ABCC8*/*KCNJ11 *genes. The presence of genetic variations in the *ABCC8 *and *KCNJ11 *genes, which are responsible for encoding components of the potassium channel found in beta cells, has the potential to impact the individual’s response to sulfonylurea therapy. Certain variants have been found to be correlated with increased susceptibility to hypoglycemia, whereas other variants have been observed to potentially diminish the efficacy of the medication [15]. The utilization of pharmacogenomics testing can assist in the identification of the most appropriate sulfonylurea or alternative therapies for individual patients.

Dipeptidyl peptidase-4 inhibitors and their relationship with the *DPP4 *gene: Dipeptidyl peptidase-4 (DPP-4) inhibitors are a class of oral medications that enhance glycemic control by impeding the degradation of incretin hormones. Genetic variations in the *DPP4 *gene have the potential to impact the enzymatic activity of DPP-4, thereby influencing the individual’s response to DPP-4 inhibitors. The utilization of pharmacogenomics-guided therapy can aid in the identification of patients who may derive greater benefits from this particular class of medications or those who may achieve improved outcomes through alternative treatment options [16].

Warfarin and the influence of *CYP2C9*/*VKORC1 *genes: Although not primarily intended as a diabetes treatment, warfarin is frequently prescribed to individuals with diabetes to mitigate the risk of thrombosis. The presence of genetic variations in the *CYP2C9 *and *VKORC1 *genes has the potential to impact both the metabolism and sensitivity of warfarin. Pharmacogenomics testing can be utilized to ascertain the optimal warfarin dosage for diabetic patients, with the aim of mitigating the potential for bleeding while still maintaining the desired anticoagulation effects [17]. Pharmacogenomics offers a valuable tool for optimizing diabetes management through the customization of drug therapies to suit individual patients. Clinicians can enhance treatment plans, enhance drug efficacy, and decrease the probability of side effects by taking into account genetic variations that impact drug response and adverse reactions. With the continuous progress of pharmacogenomics research, there is an anticipation that it will bring about a transformative impact on diabetes care. This will result in the development of treatment approaches that are more tailored and efficient, catering to the individual needs of patients with diabetes [18].

Lifestyle interventions and behavior modification in diabetes mellitus

Lifestyle interventions and behavior modification are essential components in the management of diabetes mellitus, a chronic metabolic disorder characterized by elevated levels of blood glucose. The non-pharmacological approaches discussed here are designed to provide patients with the necessary knowledge and skills to make beneficial lifestyle changes that can have a substantial impact on their diabetes management and overall health [19]. Customized lifestyle interventions, specifically designed to meet the unique needs and preferences of individual patients, have shown encouraging results in the management of diabetes.

Influence of Personalized Lifestyle Interventions on Diabetes Management

Personalized lifestyle interventions have demonstrated significant efficacy in multiple facets of diabetes management. By tailoring dietary plans, providing physical activity recommendations, and developing comprehensive treatment strategies for individual patients, these interventions effectively target specific challenges and barriers that are distinct to each person. This approach effectively improves treatment adherence, fosters long-term behavior change, and enhances patient engagement in self-management. Tailored dietary plans, such as carbohydrate counting and meal plans based on glycemic index, have been shown to assist patients in achieving improved glycemic control [20]. Customizing these plans to accommodate the unique food preferences and cultural backgrounds of individuals facilitates the adoption and maintenance of healthier dietary habits among patients. In addition, the implementation of personalized physical activity recommendations serves to ensure that patients participate in exercises that they find enjoyable and are able to perform, resulting in enhanced glucose regulation. Obesity is a notable risk factor for the development of type 2 diabetes and has the potential to exacerbate complications associated with diabetes [12-14]. Tailored lifestyle interventions aim to address weight management by implementing personalized strategies such as individualized calorie restriction, portion control, and physical activity plans. Research has indicated that implementing personalized strategies leads to increased effectiveness in achieving weight loss goals and improved long-term weight management outcomes, as opposed to utilizing generic interventions. Diabetes can potentially exert a psychological toll on individuals, manifesting as stress, anxiety, and depression. Personalized lifestyle interventions frequently incorporate behavior modification techniques, such as cognitive-behavioral therapy (CBT) and motivational interviewing, to effectively address emotional obstacles and enhance psychological well-being. By providing personalized assistance and counseling, individuals are better prepared to handle the difficulties associated with managing their diabetes [15-18].

Utilization of Behavior Modification Techniques for the Enhancement of Treatment Adherence

The utilization of behavior modification techniques plays a crucial role in improving treatment adherence and facilitating long-term behavior change in the management of diabetes. These techniques prioritize the empowerment of patients in developing self-efficacy, establishing realistic goals, and embracing positive health behaviors. Facilitating a collaborative process to establish goals that are both realistic and attainable with patients is essential in promoting their active engagement in the management of their diabetes. These objectives may include implementing dietary modifications, establishing exercise regimens, and ensuring adherence to medication protocols [21]. By decomposing larger objectives into smaller, achievable steps, individuals are more inclined to comply with their treatment plans. Self-monitoring is an essential practice that involves regularly assessing blood glucose levels, monitoring food intake, and tracking physical activity. This practice promotes self-awareness and fosters a sense of personal responsibility. Research has shown that individuals who diligently monitor their progress are more inclined to recognize recurring patterns and subsequently make well-informed decisions to enhance their management of diabetes. CBT is a therapeutic approach that assists individuals in recognizing and altering detrimental thought patterns and behaviors associated with the management of diabetes [22]. By effectively addressing the underlying psychological issues, CBT has the potential to enhance treatment adherence and empower patients to embrace healthier lifestyles.

Motivational interviewing (MI) is a patient-centered approach that aims to elicit individuals’ intrinsic motivations for making positive changes in their lives. The platform enables effective communication and collaboration between patients and healthcare providers, fostering adherence and engagement in diabetes self-care [23]. Encouraging patient participation in support groups or incorporating family members into the care process offers valuable emotional support and serves to strengthen positive behaviors. Having a robust social support system is crucial for maintaining behavior change over an extended period.

Research on Personalized Dietary Plans and Physical Activity Recommendations

Numerous studies have been conducted to examine the effects of personalized dietary plans and physical activity recommendations on the management of diabetes. Customized dietary plans, such as low-carbohydrate diets and Mediterranean diets, have exhibited noteworthy enhancements in glycemic control, weight reduction, and reduction of cardiovascular risk among individuals with diabetes. Low-carbohydrate diets have been the subject of several studies, which have indicated that these diets, by reducing the consumption of high-glycemic carbohydrates, may result in improved glycemic control and weight loss among individuals diagnosed with type 2 diabetes [24]. These dietary plans prioritize the consumption of nutrient-dense foods, such as proteins, healthy fats, and non-starchy vegetables. These food choices have been linked to enhanced insulin sensitivity and lowered blood glucose levels. The Mediterranean diet is recognized for its emphasis on consuming ample amounts of fruits, vegetables, whole grains, nuts, and olive oil. This dietary pattern has been associated with a multitude of health advantages, such as a decreased likelihood of developing cardiovascular diseases and improved glycemic control. Research has demonstrated that this specific dietary pattern can be customized to suit individual preferences, all the while delivering substantial health advantages for individuals diagnosed with diabetes [25]. Research has been conducted to examine the efficacy of personalized physical activity recommendations, taking into account variables such as age, level of fitness, and complications related to diabetes. Customized exercise plans have demonstrated the ability to enhance physical fitness, optimize glucose regulation, and mitigate cardiovascular risk factors in individuals diagnosed with diabetes.

Lifestyle interventions and behavior modification are integral aspects of diabetes management in conjunction with pharmacological therapies. Tailored lifestyle interventions, taking into account the unique needs and preferences of individual patients, have shown notable efficacy in enhancing glycemic control, managing weight, and promoting overall well-being. The implementation of behavior modification techniques serves to improve treatment adherence, thereby empowering patients to establish enduring lifestyle changes and maintain favorable health behaviors [26]. Research on personalized dietary plans and physical activity recommendations provides evidence for the effectiveness of customized approaches in improving diabetes outcomes. By integrating these individualized strategies into diabetes care, healthcare providers can empower patients to actively participate in their self-management and attain enhanced quality of life.

Telemedicine and remote patient monitoring in diabetes management

Telemedicine and remote patient monitoring have become influential tools in contemporary healthcare, fundamentally transforming the delivery of healthcare services, particularly in the realm of diabetes management. Through the utilization of technology, telemedicine facilitates healthcare providers in conducting remote assessments, diagnoses, and treatments of patients, thereby delivering individualized care and enhancing patient outcomes [26-29]. The integration of remote patient monitoring, along with virtual consultations, effectively enhances patient engagement by empowering individuals to actively participate in their diabetes self-management. Nevertheless, telemedicine presents significant potential in the field of diabetes management, yet it is crucial to acknowledge and tackle the challenges it encounters to fully harness its benefits [30].

Significance of telemedicine in providing individualized diabetes care

The implementation of telemedicine has had a notable influence on the provision of individualized diabetes care by eliminating obstacles to accessibility and enabling patients to receive prompt and convenient medical assistance. Telemedicine enables healthcare providers to remotely monitor blood glucose levels, make necessary adjustments to treatment plans, and deliver timely feedback, thereby customizing interventions to meet the unique needs of each patient.

Remote Monitoring of Blood Glucose

Through the incorporation of CGM systems and intelligent devices, healthcare professionals are able to remotely monitor blood glucose levels [30]. This enables prompt modifications in treatment regimens according to individual glycemic patterns, thereby optimizing glycemic control and mitigating the risk of complications associated with diabetes. Telemedicine platforms facilitate the delivery of customized treatment plans that take into account the distinct medical history, lifestyle, and preferences of each patient. These customized plans enable patients to actively engage in their diabetes management and adhere to the prescribed therapies [30]. Telemedicine enables the provision of remote lifestyle coaching services, allowing healthcare providers to deliver personalized dietary guidance, exercise suggestions, and behavior modification strategies. The customized approach implemented in this context effectively targets specific obstacles to adherence and promotes the active involvement of patients in managing their diabetes.

Enhancing Patient Engagement Via Remote Patient Monitoring and Virtual Consultations

Remote patient monitoring and virtual consultations are essential components in enhancing patient engagement and enabling individuals to actively participate in the management of their diabetes. Through the utilization of remote patient monitoring, patients have the ability to receive immediate feedback regarding their blood glucose levels and lifestyle behaviors. The provision of prompt feedback facilitates the development of self-awareness and enables patients to make timely adjustments to their management strategies. Virtual consultations offer a solution to the challenges posed by in-person visits, enhancing the accessibility of healthcare services [30]. This is particularly beneficial for patients residing in remote areas or experiencing mobility limitations. The convenience offered by virtual visits promotes regular follow-ups and enhances communication between patients and healthcare providers. Remote patient monitoring platforms frequently incorporate educational resources and tools that provide patients with valuable information regarding the management of diabetes [25-30]. The provision of educational materials facilitates patients’ comprehension of their medical condition and empowers them to make well-informed choices regarding their healthcare.

Challenges and Future Potential of Telemedicine in Diabetes Management

Although telemedicine has demonstrated considerable potential in the field of diabetes management, there exist several challenges that must be effectively addressed to facilitate its widespread adoption and ensure its optimal utilization. Technological barriers pose a challenge as certain patients lack access to essential technology or reliable internet connections, thereby restricting their capacity to effectively participate in telemedicine. It is imperative to address the digital divide to establish fair and equal access to telemedicine services. The transmission and storage of patient data in telemedicine platforms give rise to concerns regarding data security and privacy [18]. The implementation of robust data protection measures is of utmost importance to ensure the security and protection of patient information. It is imperative for healthcare providers to receive comprehensive training to proficiently utilize telemedicine tools and deliver exceptional virtual care services. Ongoing education and skills development are crucial to facilitate the smooth integration of telemedicine into the field of diabetes management. Telemedicine encounters regulatory and reimbursement challenges due to the diverse regulations and reimbursement policies that exist across different geographical areas [20]. It is imperative to streamline the existing regulations and establish equitable reimbursement policies for telemedicine services to promote their extensive implementation.

In summary, the utilization of telemedicine and remote patient monitoring has significantly transformed the field of diabetes management. These technologies have effectively facilitated the delivery of personalized care, fostered greater patient engagement, and ultimately led to improved health outcomes [20-22]. Telemedicine enables patients to actively participate in their diabetes self-management by utilizing remote monitoring of blood glucose levels and personalized treatment plans. Virtual consultations and remote lifestyle coaching offer enhanced convenience and accessibility, thereby facilitating improved communication between patients and healthcare providers. Despite the inherent challenges posed by technology, data security, and regulatory concerns, the future prospects of telemedicine in the realm of diabetes management exhibit considerable promise. By effectively addressing these challenges and fully utilizing the capabilities of telemedicine, healthcare providers have the opportunity to enhance the quality of care and enable patients to take charge of their health, leading to improved outcomes for individuals with diabetes [20-25].

Challenges and limitations in diabetes mellitus management

The management of diabetes mellitus poses various challenges and limitations that affect the integration of personalized medicine into clinical practice. Despite the progress made in technology and medical research, these challenges present substantial obstacles in attaining the best possible results for patients diagnosed with diabetes. It is imperative to address these challenges to enhance diabetes care and optimize the potential of personalized medicine in effectively managing this intricate chronic condition. Diabetes mellitus is a multifaceted condition characterized by diverse subtypes and underlying pathophysiological mechanisms [19]. The intricate nature of this complexity presents a significant challenge in the development of precise and universally applicable personalized treatment strategies for every patient. While genetic testing and biomarker analysis have the potential to provide valuable insights for personalized approaches, it is important to note that the integration of this information into clinical decision-making necessitates additional research and validation [20]. Personalized medicine frequently entails the utilization of sophisticated technologies and resources, including genetic testing, CGM, and wearable devices. However, it is important to note that not all patients have equitable access to these technologies, especially in low-resource settings or among disadvantaged populations [21]. The challenge of ensuring equitable access to personalized medicine in diabetes management persists. The process of gathering and analyzing extensive patient data, encompassing genetic information, lifestyle factors, and medical history, can pose a significant challenge for healthcare providers. The process of integrating diverse datasets and extracting meaningful insights to inform personalized treatment plans necessitates the use of advanced informatics tools and the expertise of skilled analysts [20-24].

Ethical Considerations and Patient Privacy Concerns

The implementation of personalized medicine in diabetes management gives rise to ethical considerations concerning the acquisition of informed consent from patients. It is imperative for patients to possess a comprehensive understanding of the advantages, drawbacks, and potential ramifications associated with the disclosure of their genetic and personal information for making personalized treatment decisions [25]. The collection and storage of patients’ genetic and health-related data in the field of personalized medicine may present potential privacy risks. It is imperative to implement robust data security measures and strictly adhere to patient privacy standards to safeguard sensitive information from unauthorized access and breaches. Patients may express concerns regarding the ownership of their data and the manner in which their information will be utilized in research or disclosed to third parties. Effective and open communication, as well as well-defined policies pertaining to data sharing and ownership, play a vital role in establishing trust between patients and healthcare providers [25-30].

Potential Areas for Further Research and Improvement

Further investigation is required to enhance our comprehension of the genetic elements that contribute to the onset and advancement of diabetes. The integration of genetic data with clinical information has the potential to identify distinct patient subgroups that exhibit varying responses to specific treatments. This advancement holds promise for the development of more precise and targeted therapeutic approaches [21-23]. The development of robust risk prediction models utilizing diverse patient data can significantly contribute to the early detection and prevention of complications associated with diabetes. The process of accurately assessing risks can provide valuable guidance for making personalized treatment decisions and ultimately maximizing outcomes. The importance of conducting research on effective strategies for patient education and empowerment cannot be overstated [22-24]. Such research is crucial in providing patients with the necessary tools and knowledge to make informed decisions regarding their treatment plans and lifestyle adjustments. Enhancing patient engagement is crucial for achieving effective personalized diabetes management. The integration of personalized medicine into the broader healthcare system necessitates the establishment of alignment with existing clinical workflows and electronic health record systems. The development of interfaces that are user-friendly and decision-support tools can greatly enhance the integration of personalized approaches into routine clinical practice [24-26].

In summary, personalized medicine exhibits significant potential in the management of diabetes mellitus; however, it is important to acknowledge the challenges and limitations in its implementation. The intricate nature of diabetes, challenges related to technology and resource availability, and the need for effective data integration and interpretation present significant obstacles to achieving widespread implementation [26]. It is imperative to address ethical considerations pertaining to informed consent, data security, and patient privacy to establish and maintain patient trust. Future research should prioritize the refinement of risk prediction models, the integration of genetic data, and the enhancement of patient education and empowerment [28]. Through the successful navigation of these challenges and the cultivation of ongoing enhancements, personalized medicine has the potential to assume a progressively influential position in the optimization of diabetes care and the enhancement of patient outcomes.

## Conclusions

This literature review pertaining to personalized medicine in the management of diabetes sheds light on the potential advantages and obstacles associated with this approach. The implementation of personalized medicine has the potential to enhance glycemic control, foster patient engagement, and optimize treatment selection by customizing healthcare plans based on individual genetic, lifestyle, and clinical factors. The implementation of this approach is of utmost importance given the complex nature of diabetes, wherein a standardized approach may not yield optimal results for every individual. Advanced technologies such as genetic testing, CGM, and telemedicine facilitate the active involvement of patients and support well-informed decision-making. To ensure successful implementation, it is recommended that future research prioritize conducting long-term efficacy studies, integrating healthcare systems, fostering collaboration among stakeholders, enhancing patient education, and addressing ethical considerations. By prioritizing these areas, it is possible to facilitate a revolution in diabetes management, resulting in enhanced patient outcomes and a more accurate and streamlined approach to healthcare.
